# Supplementary material for: Chemomechanical modification of quantum emission in monolayer WSe2
Source: Nat Commun. 2023 Apr 17;14:2193. doi: 10.1038/s41467-023-37892-0 (PMC10110606; doi:10.1038/s41467-023-37892-0)
Supplement: Supplementary file 1 — Supplementary Information [file 41467_2023_37892_MOESM1_ESM.docx]

**Supplementary Information**

**Chemomechanical Modification of Quantum Emission in Monolayer WSe_2_**

M. Iqbal Bakti Utama^1,10^, Hongfei Zeng^2,10^, Tumpa Sadhukhan^3,9,10^, Anushka Dasgupta^1,10^, S. Carin Gavin^2^, Riddhi Ananth^3^, Dmitry Lebedev^1^, Wei Wang^4^, Jia-Shiang Chen^4,5^, Kenji Watanabe^6^, Takashi Taniguchi^7^, Tobin J. Marks^1,3^, Xuedan Ma^4,5^, Emily A. Weiss^3^, George C. Schatz^3,*^, Nathaniel P. Stern^2,*^, and Mark C. Hersam^1,3,8,*^

^1^Department of Materials Science and Engineering and the Materials Research Center, Northwestern University, Evanston, Illinois 60208, USA.

^2^Department of Physics and Astronomy, Northwestern University, Evanston, Illinois 60208, USA.

^3^Department of Chemistry and the Materials Research Center, Northwestern University, Evanston, Illinois 60208, USA.

^4^Center for Nanoscale Materials, Argonne National Laboratory, Lemont, Illinois 60439, USA.

^5^Northwestern-Argonne Institute of Science and Engineering, Northwestern University, Evanston, Illinois 60208, USA.

^6^Research Center for Functional Materials, National Institute for Materials Science, 1-1 Namiki, Tsukuba 305-0044, Japan.

^7^International Center for Materials Nanoarchitectonics, National Institute for Materials Science, 1-1 Namiki, Tsukuba 305-0044, Japan.

^8^Department of Electrical and Computer Engineering, Northwestern University, Evanston, IL 60208, USA.

^9^Present address: Department of Chemistry, SRM Institute of Science and Technology, Kattankulathur, Tamil Nadu 603203, India.

^10^These authors contributed equally: M. Iqbal Bakti Utama, Hongfei Zeng, Tumpa Sadhukhan, Anushka Dasgupta.

^*^e-mail: [m-hersam@northwestern.edu](mailto:m-hersam@northwestern.edu), [n-stern@northwestern.edu](mailto:n-stern@northwestern.edu%20%20edu), [g-schatz@northwestern.edu](mailto:g-schatz@northwestern.edu)


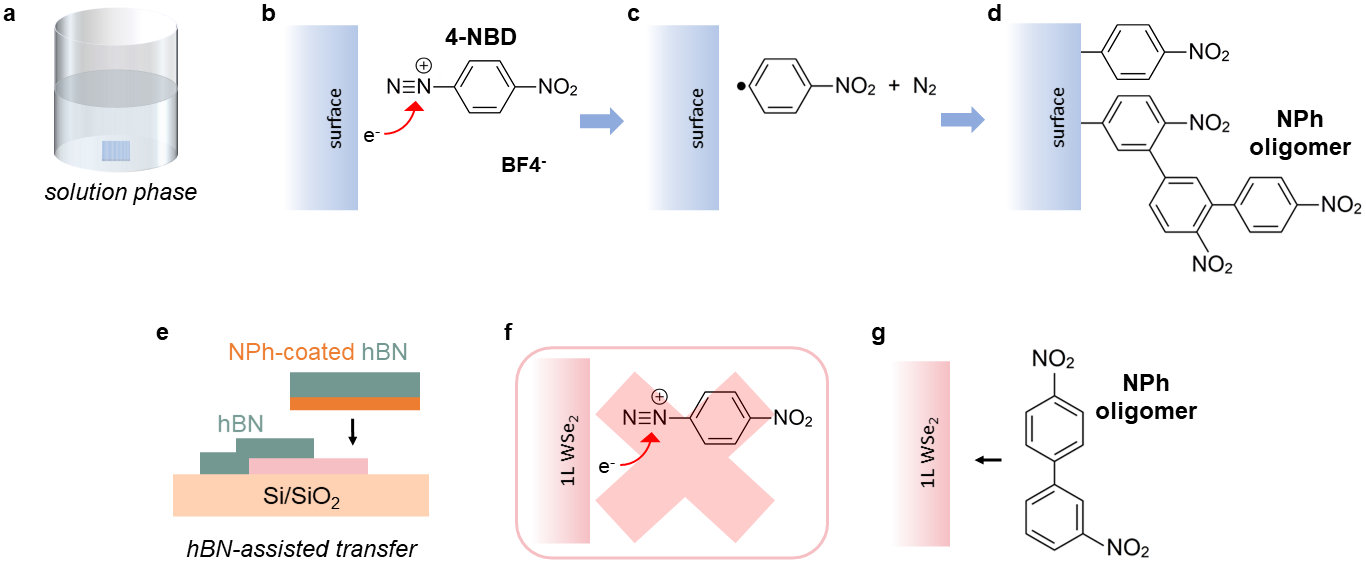


**Supplementary Fig. 1 | Functionalization scheme of nitrophenyl (NPh).** **a-d,** Solution-phase functionalization process as is typically described in the literature, which assumes a covalent bond formation between the nitrophenyl and the surface of a sample. **(a)** Schematic of solution-phase functionalization. **(b)** For the functionalization of a sample in a solution of 4-nitrobenzenediazonium (4-NBD) tetrafluoroborate (BF_4_), the 4-NBD anion withdraws electrons from the sample. **(c)** The withdrawn electron induces the formation of a diazonium radical. (d) The diazonium radical can then bond covalently to the surface as nitrophenyl (NPh) or bond to each other to form NPh oligomers. In our experiments, the NPh oligomers are found to be primarily physisorbed to the WSe_2_ surface instead of forming covalent bonds. **e-g,** hBN-assisted NPh transfer. **(e)** Schematic of the transfer process, which is detailed in Supplementary Fig. 20. **(f)** Because the monolayer WSe_2_ is not exposed to the 4-NBD ion directly, the electron withdrawal process from WSe_2_ will not occur. **(g)** Instead, the monolayer WSe_2_ is contacted directly with the NPh oligomer film with the assistance of an hBN flake, ensuring a noncovalent interaction between the NPh film and the WSe_2_ surface


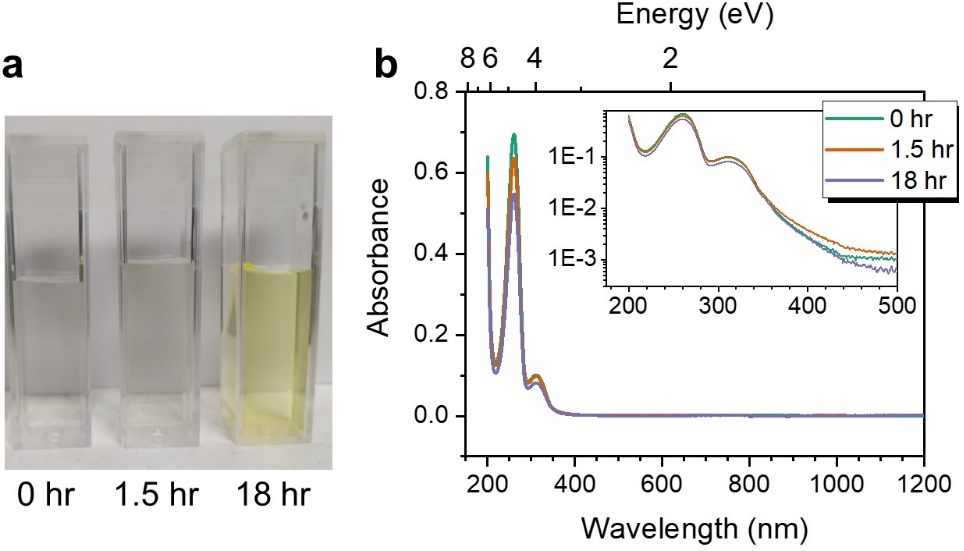


**Supplementary Fig. 2 | Aqueous solution of nitrobenzene diazonium (4-NBD). a,** Photograph of 5 mM solutions of 4-NBD: 0 hour (as-prepared), after 1.5 hour of aging under ambient conditions, and after 18 hours. Progressive formation of a yellowish tint is observed. **b,** UV-vis-NIR spectra of the 4-NBD solution at different aging times. The solution is diluted in deionized water (1:250 ratio) prior to measurement. Inset: The same data in semi-log scale. No major changes in the spectrum were observed with the aging of the solution, indicating that the solution does not decompose^1^ within the typical duration of sample immersion of 1-2 hours.


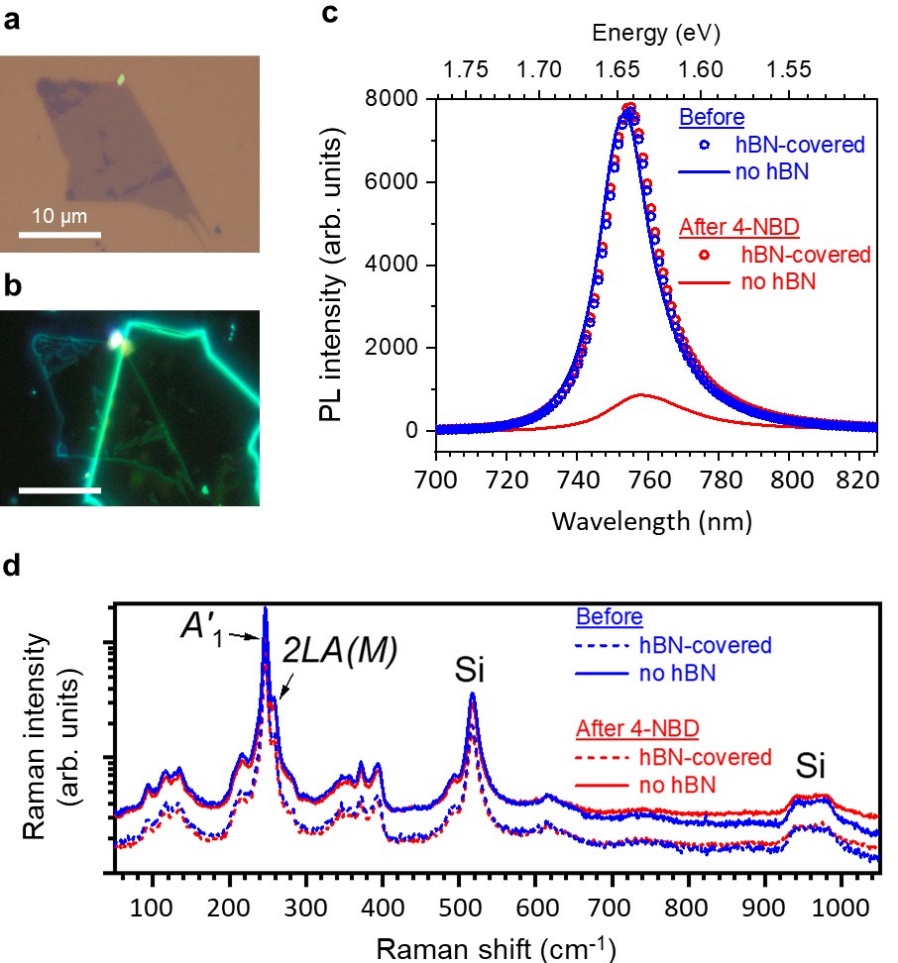


**Supplementary Fig. 3 | Characterization of 1L WSe_2_ before and after 4-NBD treatment.** This figure is based on the same sample as shown in Fig. 1 in the main text. **a,** Bright-field micrograph of as-exfoliated 1L WSe_2_. The darker patches on the flake are regions of bilayer WSe_2_. **b,** Dark-field micrograph of the sample following the hBN transfer. **c,** The raw, unnormalized spectra from Fig. 1c in the main text. For clarity in visualizing the graph, the spectrum from the hBN-covered location is reduced by showing only 1 point for every 5 data points. **d,** Raman spectra before (dashed lines) and after 4-NBD treatment (solid lines) at two different locations: a region covered with hBN (blue) and without the hBN cover (red). No new spectral features or significant shifts of the Raman modes were observed following the 4-NBD treatment, indicating that 1L WSe_2_ does not experience structural changes with the 4-NBD treatment.


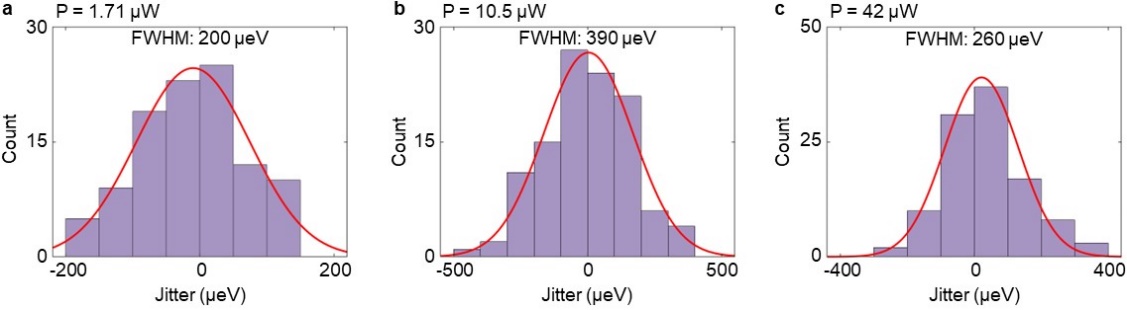


**Supplementary Fig. 4 | Spectral wandering of a chemomechanically modified emitter.** The histograms are constructed based on time series measurements of the emission spectrum of the emitter shown in Fig. 2 in the main text. **a-c,** The histograms of emitter peak position for excitation power used in the measurements are: **(a)** 1.71 µW, **(b)** 10.5 µW, and **(c)** 42 µW. The data were collected with a time bin of 0.5 s from ~110 consecutive spectra. We fitted the distribution with a Gaussian function, resulting in FWHM of 200, 390, and 260 µeV for the different laser powers. This level of jittering is a factor of ~2 larger than previous reports of monolayer WSe_2_ on nanopillar^2^, but is still an order of magnitude smaller than other reports on randomly occurring emitters from WSe_2_ on SiO_2_ substrates^3,4^.

**
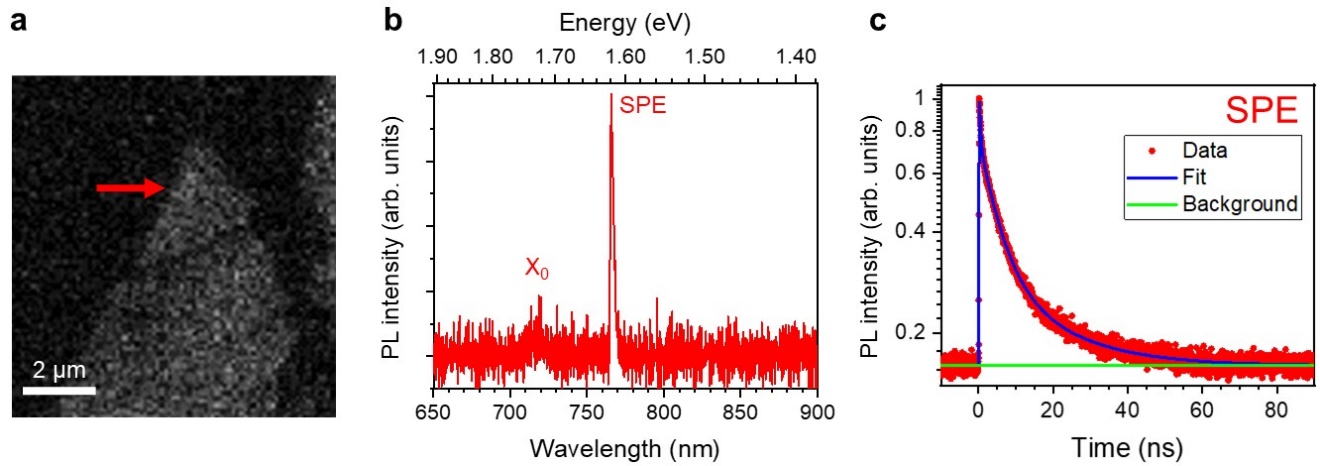
**

**Supplementary Fig. 5 | PL lifetime measurements of SPE following the 4-NBD treatment. a,** Photoluminescence (PL) image of a functionalized 1L WSe_2_ flake on a flat SiO_2_ substrate (same sample as discussed in Fig. 2 of the main text). The red arrow indicates the location of the wrinkle where the SPE emerged from the sample. We note that the SPE wavelength has shifted to ~760 nm during this measurement run following several heating and cooling cycles in the optical cryostat. **b,** Steady-state PL spectrum with the neutral exciton (X_0_) and SPE indicated. **c**, Time-resolved PL intensity of the SPE. The blue curve is the best fit to the experimental data (red points) using the function $I\left( t \right)=\sum_{i} A_{i}e^{\left( -\frac{t}{\tau_{i}} \right)}$, where $\tau_{i}$ and $A_{i}$ are the radiative lifetimes and their weighted contributions, respectively. The fitted parameters are provided in Supplementary Table 1.

**Supplementary Table 1 | Fitted parameters from the time-resolved measurements.** The fitted lifetimes ($\tau_{i}$) and weighted contribution ($A_{i}$) of the SPE based on the data in Supplementary Fig. 5 are shown. The averaged lifetime can be estimated as $\left\langle t \right\rangle={\sum\left( A_{i}{t_{i}}^{2} \right)}/{\sum\left( A_{i}t_{i} \right)=}$ 11.7 ns. These PL lifetimes are consistent with SPE typically observed from WSe_2_ in the literature^5,6,7^. In contrast, emission from strain-confined interlayer exciton species have been reported to have lifetimes as long as microseconds^8^.

**
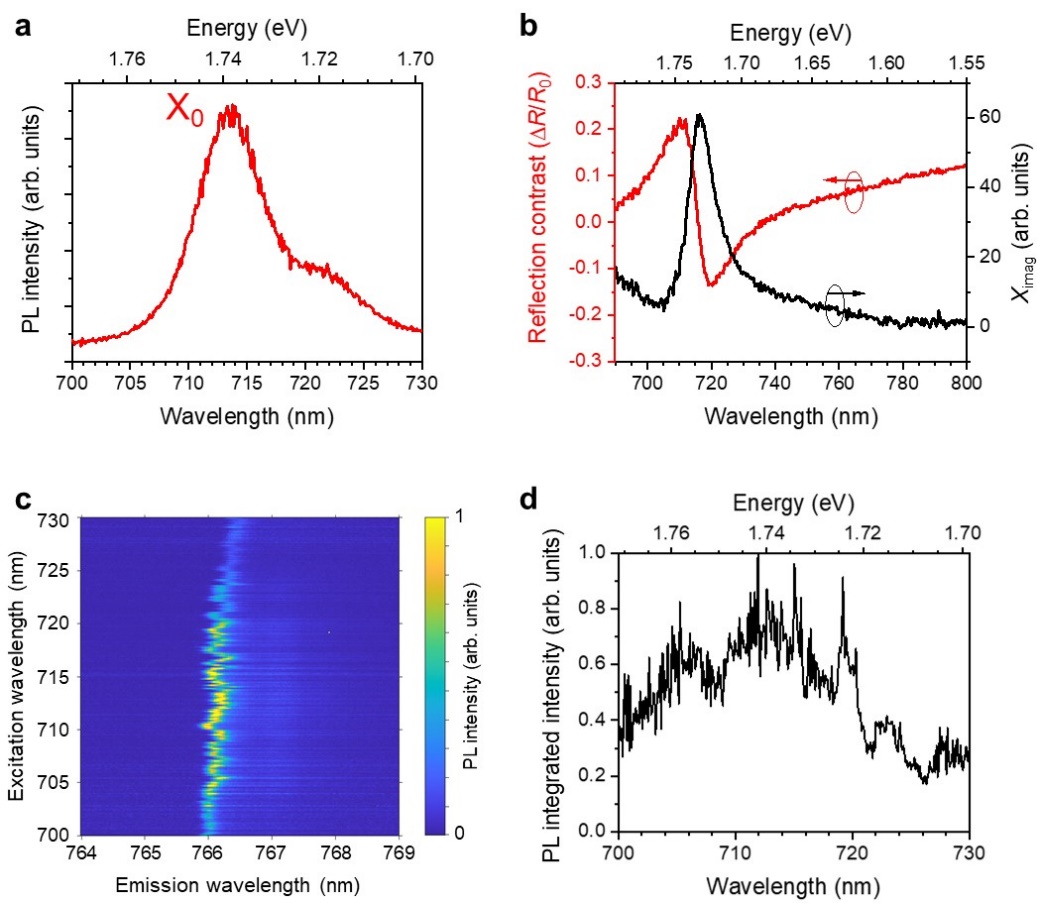
**

**Supplementary Fig. 6 | WSe_2_ exciton resonance and photoluminescence excitation of SPE. a,** The PL spectrum of a flat WSe_2_ monolayer after 4-NBD treatment around the neutral A exciton feature (X_0_) that is centered at ~714 nm (same spectrum as in Fig. 2b in the main text). **b,** Reflection contrast of the sample after 4-NBD treatment (red curve). The imaginary part of susceptibility ($\chi_{\mathrm{imag}}$, black curve) estimated using a Kramers-Kronig-constrained analysis^9^ of the reflection contrast indicates an excitonic absorption peak from X_0_ centered at ~715 nm. **b,** PLE spectra of a chemomechanically-modified SPE. **d,** Integrated intensity plot with respect to excitation wavelength shows an enhancement as the excitation is swept through the X_0_ exciton resonance, suggesting a resonance enhancement behavior.


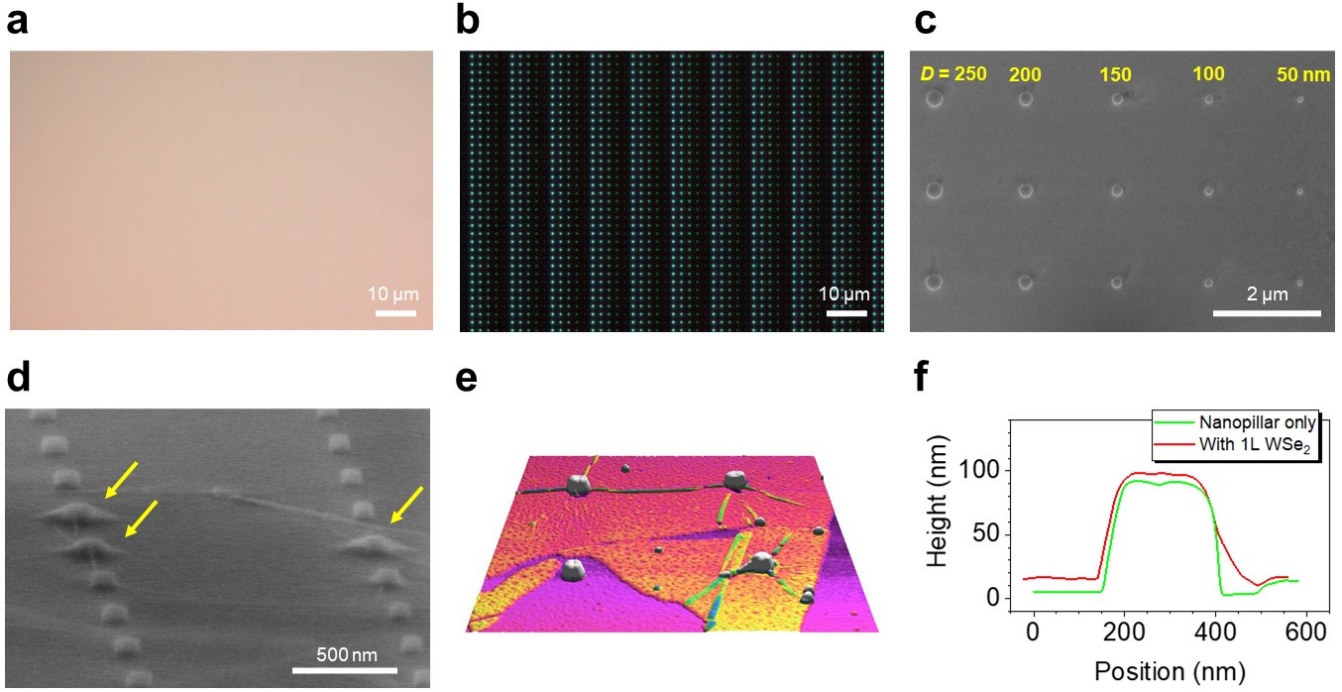


**Supplementary Fig. 7 | SiO_x_ nanopillars. a,b,** Micrographs of the SiO_x_ nanopillar array fabricated by electron-beam lithography on a Si substrate with 300 nm thick SiO_2_, as viewed in **(a)** bright-field and **(b)** dark-field images. The nanopillar cannot be seen clearly in the bright-field image but can be observed in the dark-field image. The array consists of repeating rows of nanopillars with diameters of 250, 200, 150, 100, and 50 nm. The height of the nanopillars is approximately 90-100 nm. Significant differences were not observed in the spectra using nanopillars with different diameters. **c,** Top-view scanning electron microscopy (SEM) image of the nanopillars. The nanopillar diameter (*D*) is indicated. **d,** SEM image of the nanopillar array after transfer of a 1L WSe_2_ flake. Yellow arrows mark the nanopillar with the strained monolayer. **e,** Atomic force microscopy (AFM) image of the sample in Fig. 3 following the 4-NBD treatment. The nanopillar at the bottom right is the position P1. **f,** The height profile of a bare nanopillar (green curve) and a nanopillar that supports 1L WSe_2_ after the transfer (red curve).

**
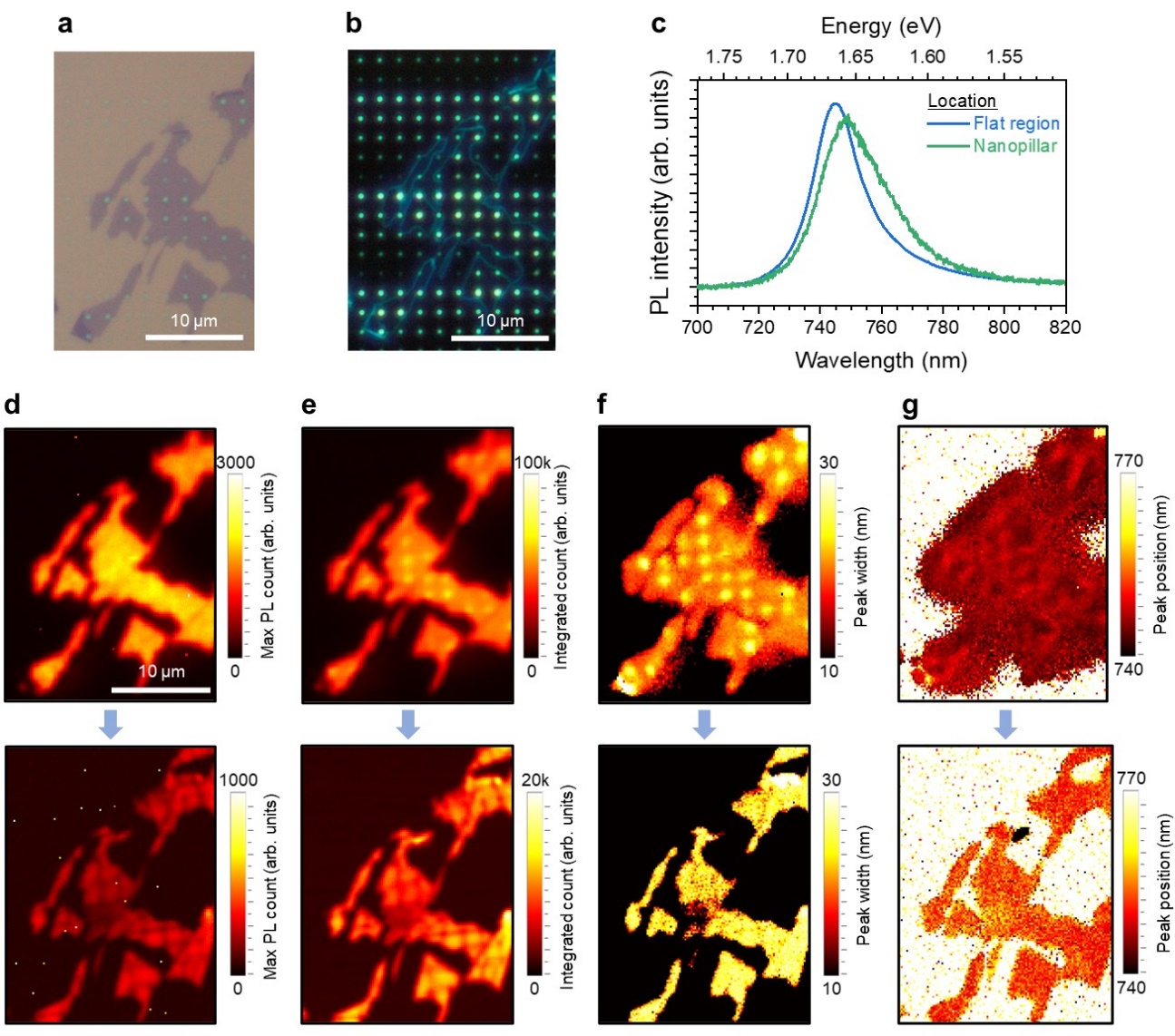
**

**Supplementary Fig. 8 | Room temperature PL map of 1L WSe_2_ on a nanopillar array before and after 4-NBD treatment. a,b,** Optical micrograph of the sample in bright-field **(a)** and dark-field **(b)** imaging modes. **c,** PL spectra of the as-transferred sample at the flat region (blue) and at the nanopillar-strained location (green). The strained location shows a broadened linewidth in agreement with trion funneling behavior. **d-g,** PL maps before (upper row) and after 4-NBD treatment (lower row). The map is presented according to **(d)** the peak PL count, **(e)** integrated PL count, **(f)** PL linewidth, and **(g)** peak position.

**
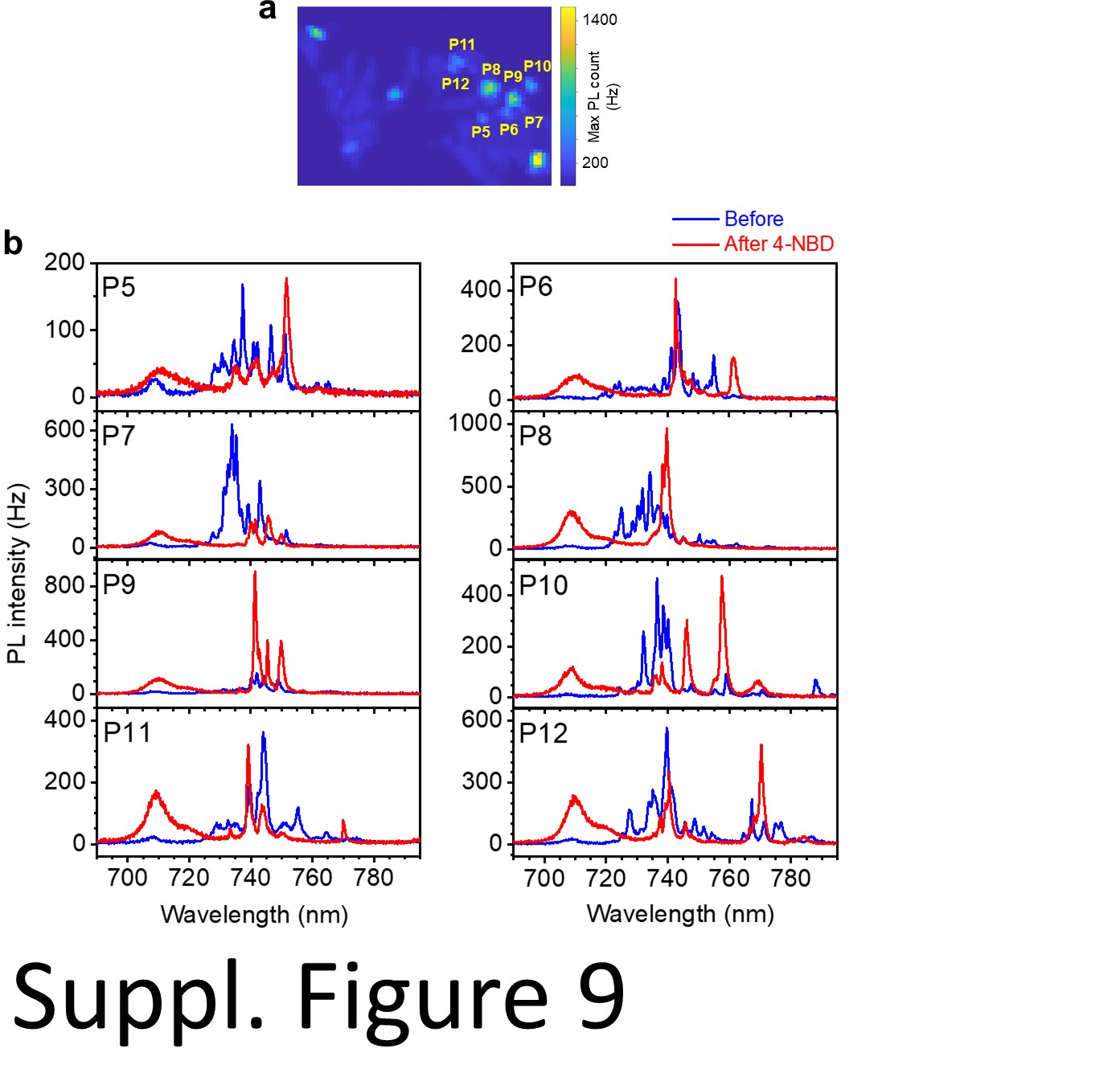
**

**Supplementary Fig. 9 | Low-temperature (T = 1.7 K) PL spectra of the chemomechanically-modified emitters. a,** Labelled map of PL peak intensity between the wavelengths of 720 nm and 800 nm only. This map is from the same dataset as Fig. 3d in the main text. **b,** PL spectra before (blue) and after 4-NBD treatment (red) at nanopillar positions P5 - P12 as labelled in (a). The excitation powers used in the measurement are 10 µW before functionalization and 45 µW after 4-NBD treatment. While the neutral exciton intensity scales proportionally with laser power, the intensity of the emitters typically saturates for excitation powers of several µW.


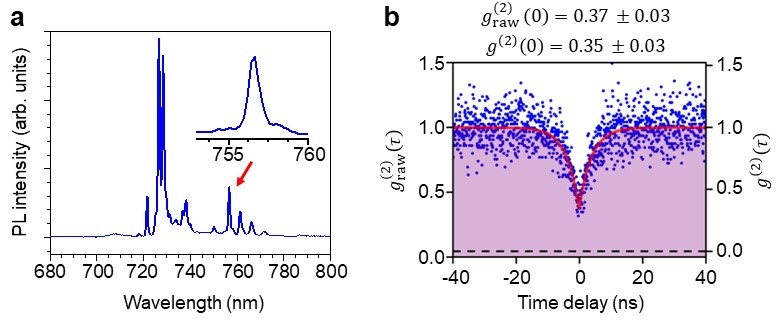


**Supplementary Fig. 10 |** **An example of** $\boldsymbol{g}^{\left( \boldsymbol{2} \right)}\left( \boldsymbol{\tau} \right)$ **measurement on a monolayer WSe_2_ flake on a nanopillar before functionalization. a,** Low temperature PL spectrum of the emitter site. The $g^{\left( 2 \right)}\left( \tau\right)$ measurement was performed on the emitter at 756 nm marked by the red arrow. Inset: Zoom in of the emitter peak. **b,** $g^{\left( 2 \right)}\left( \tau\right)$ data of the emitter at 756 nm in (a). More generally, it is not always possible to completely filter out the signal of other emitter states at nearby energy or broad defect background from the sample to the allow extraction of low $g^{\left( 2 \right)}\left( 0 \right)$ values or even clear anti-bunching signature. The use of 4-NBD treatment helps to simplify the emission spectrum and produce isolated peaks more frequently.

**
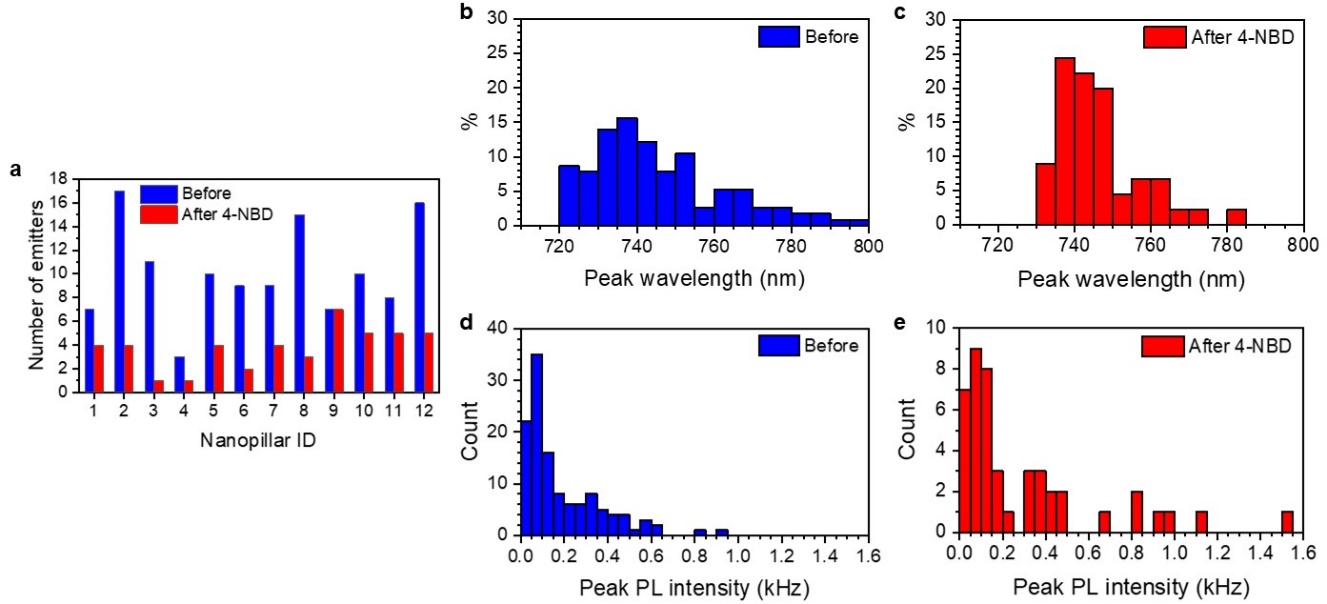
**

**Supplementary Fig. 11 | Statistics of the emitters from WSe_2_ strained on nanopillars before and after 4-NBD treatment.** These data are summarized from the spectra from positions P1-12 presented in Fig. 3f in the main text and in Supplementary Fig. 9b. **a,** Comparison of the number of emitter lines observed before and after 4-NBD treatment. In addition to the 12 nanopillar sites presented, an additional 12 nanopillar sites exist on the same sample that have their dense defect emitters completely quenched following 4-NBD treatment, leaving only features of the neutral exciton. One of the claims of our work is the use of 4-NBD treatment to simplify the low-temperature emission spectrum of WSe_2_. Along these lines, our experiments show a nearly 100% yield in the observation of fewer emitter lines after treatment with 4-NBD as compared to the pristine WSe_2_ sample at the same measurement location. Although in most cases there is more than 1 emitter observed per site, the emitters are more likely isolated from each other energetically after the emission spectrum is simplified such that it is possible to obtain low $g^{\left( 2 \right)}\left( 0 \right)$ values (e.g., Fig. 3g-h in the main text). **b,c,** Distribution of the peak wavelength from the emitters observed **(b)** before and **(c)** after 4-NBD treatment. The emitters in monolayer WSe_2_ are typically observed within a wavelength range of 720-800 nm (corresponding to a range of ~170 meV), which results from the energy range of the emissive sub-bandgap defect states in WSe_2_. After 4-NBD treatment, we observed some narrowing of the distribution, with over 60% of the emitters present after functionalization residing in a wavelength range of 735-750 nm. **d,e,** The peak PL intensity for the emitters **(d)** before and **(e)** after 4-NBD treatment, which shows comparable intensity range and distribution.


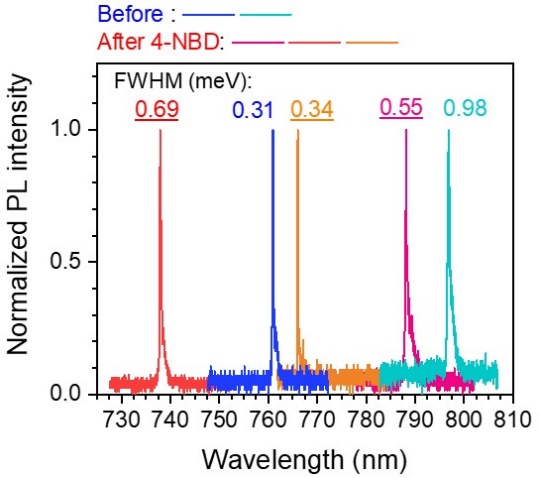


**Supplementary Fig. 12 | Sharp emitters present on strained monolayer WSe­_2_ before and after 4-NBD treatment.** The emitter peaked at ~790 nm corresponds to the sample discussed in Fig. 2c-d in the main text. It is not always feasible to extract the linewidth accurately for many of the emitters (especially for the case prior to functionalization) because the emitter lines are not energetically well-isolated. We show here instead several examples of the sharpest emitters that we have observed in our monolayer WSe_2_ samples for both before and after 4-NBD treatment cases to show the narrowest observed linewidths. The FWHM values from Gaussian fitting of the zero-phonon line are shown above each of the peaks, with the values that are underlined corresponding to emitters from samples after 4-NBD treatment. Both before and after 4-NBD treatment, we have observed quantum emitters with sub-meV linewidth down to ~0.3 meV. We remark that our functionalization scheme may also be combined with other strategies to produce even narrower emitter linewidths, such as the use of substrate materials with superior surface properties (e.g., hBN, InGaP) as compared to SiO_2_/SiO_x_ used in this work.


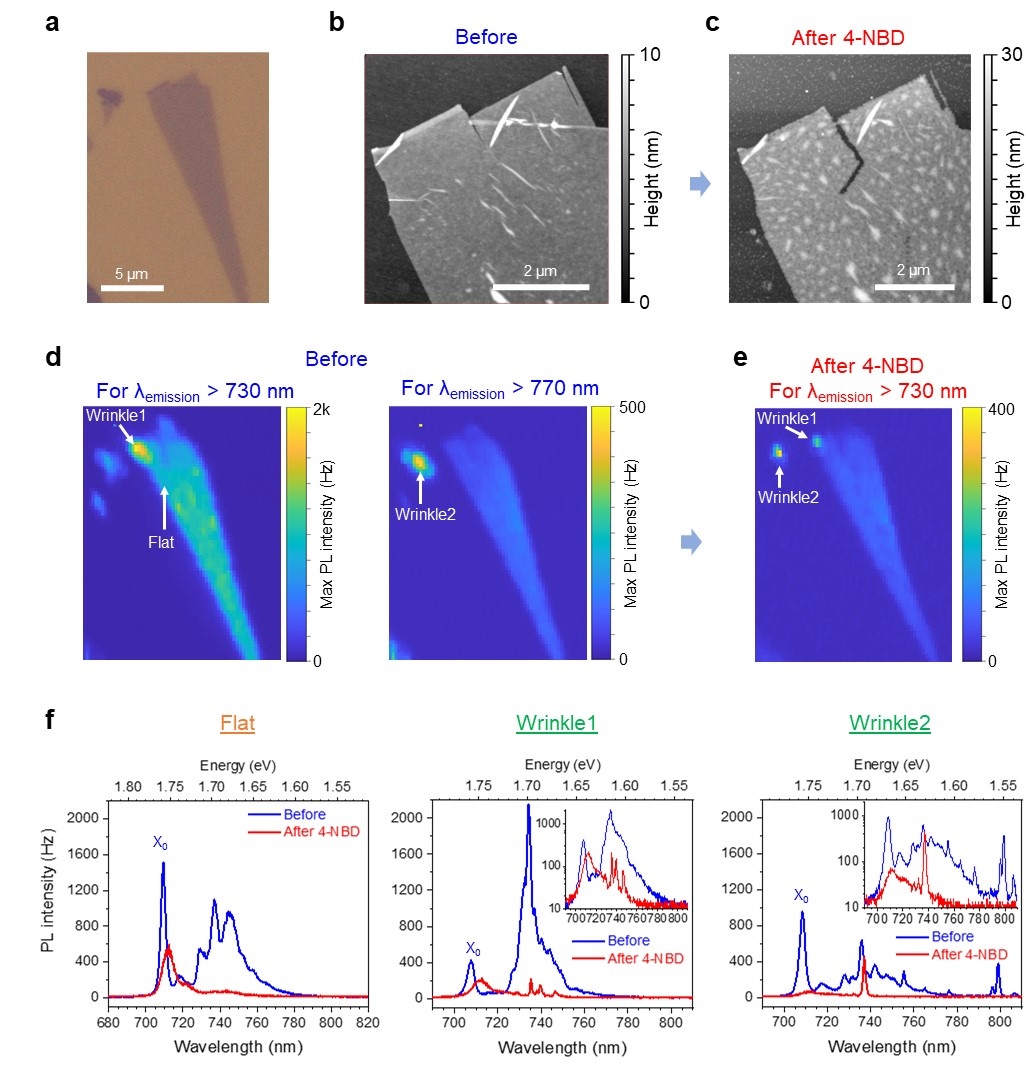


**Supplementary Fig. 13 | Additional low-temperature PL measurements on WSe_2_ monolayer flakes transferred on a Si/SiO_2_ substrate.** For both before and after 4-NBD treatment, PL measurements were performed with 635 nm CW-laser excitation at 46 µW power at T = 2.8 K (Attocube AttoDRY2100). **a,** Optical micrograph of the sample. **b,c,** AFM image of the sample **(b)** before and **(c)** after 4-NBD treatment, showing wrinkle formation at the corner of the flake. **d,** PL map of the sample before functionalization. The images show the maximum count for each pixel from the same PL map for different emission wavelength range longer than that of the neutral exciton, X_0_. (left) Emission wavelength > 730 nm. (right) Emission wavelength range > 770 nm. The bright spots from the two maps experienced enhanced defect-related emission from randomly-occurring localized strain and are labelled as “Wrinkle1” and “Wrinkle2”. **e,** PL map after 4-NBD treatment, showing the maximum PL count for emission wavelength range > 730 nm. **f,** PL spectra before and after 4-NBD treatment from the flat region of the sample (left) and locations on the map indicated as Wrinkle1 (middle) and Wrinkle2 (right). At the Wrinkle1 location, the spectrally dense defect emission lines are bundled together into a single high-intensity broad feature between 720 nm and 760 nm. The insets show the spectra in logarithmic y-axis for better visualization. Simplified emission spectra after 4-NBD treatment are observed, reducing the number of defect-related emitter lines.

The original purpose of the low-temperature PL measurements (including Fig. 3 for the sample on nanopillars) was to demonstrate the primary effect of 4-NBD treatment in the partial quenching of the defect-related emission lines, resulting in the simplified emission spectrum. Towards this end, measurements with different excitation power can be compared since the primary properties of interest are the lineshape changes of the emission spectrum. Relatedly, the higher excitation power used after 4-NBD treatment is also beneficial for confirming that the emitter lines are indeed quenched.

However, comparison of the emitter brightness (Fig. 3 and Supplementary Fig. 9) is challenging with different excitation conditions, although at power levels at or above 10 µW the emitter intensity is close to saturation. For completeness, we conducted an additional low-temperature experiment on a different sample where the measurements before and after 4-NBD treatment are performed at the same excitation power (46 µW). This experiment allows a more direct comparison of the intensity of the defect-related emitters before and after 4-NBD treatment (Supplementary Fig. 13). In this experiment, we reproduced the main result of the manuscript, namely the simplification of the emission spectrum following the 4-NBD treatment. At sample locations that experience enhanced defect-related emission due to localized strain (e.g., Wrinkle1 and Wrinkle2 in Supplementary Fig. 13d-f), the 4-NBD treatment results in partial quenching that leaves emission lines that are better isolated energetically. The remaining emitters show peak intensities of ~100-500 kHz, which is comparable to the emitters before functionalization (most visible in the Wrinkle2 location).

In the meantime, the absolute intensity of the neutral exciton peak (X­_0_) may show some variation from sample to sample. Likewise, the amount of relative quenching of X_0_ intensity before and after 4-NBD treatment may also vary from the flat region of the sample to the region of the sample with localized strain. For example, the quenching of the X_0_ peak at the location Wrinkle2 appears more dramatic than that at the flat region, despite the defect-related feature at Wrinkle2 remaining of comparable intensity. It is possible that this effect may also appear differently at different nanopillar-strained locations of the sample in such a way that the quenching amount of X_0_ may appear reduced. The variation of measured intensity of X_0_ at such strained locations is also complicated by the fact that the emission involves multiple defect-related recombination decay channels, and so detailed understanding on the dynamics will require time-resolved measurements in future work.

**
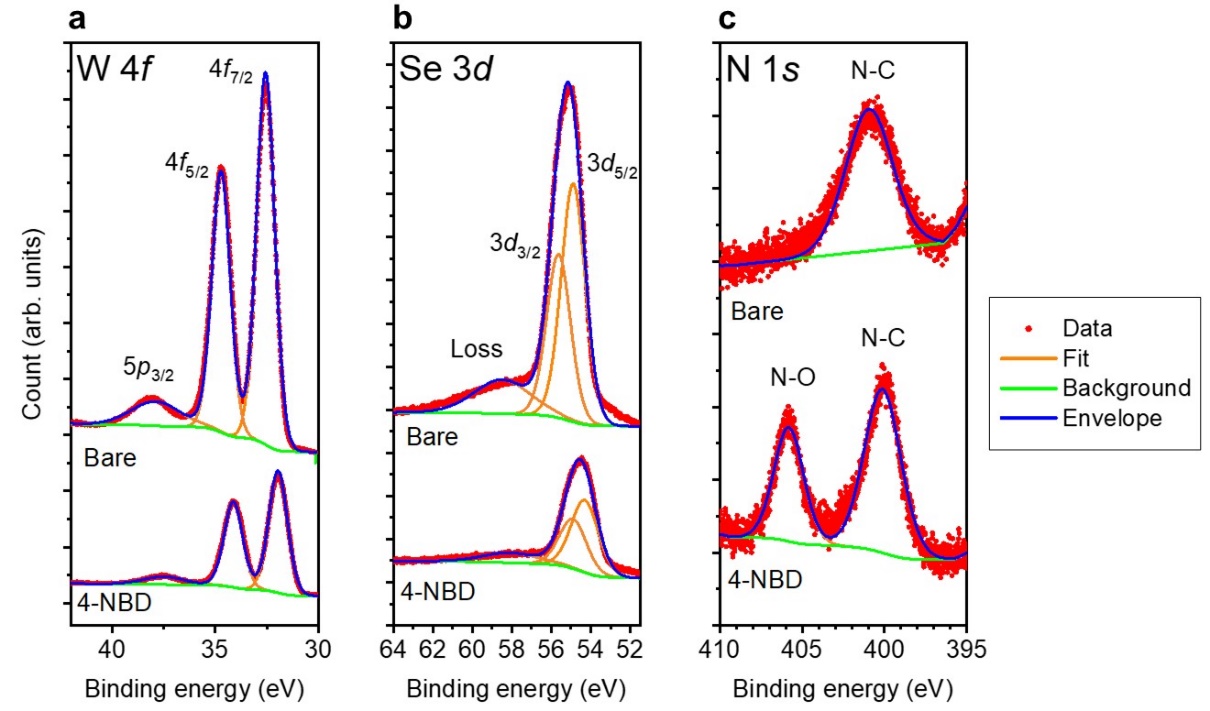
**

**Supplementary Fig. 14 | XPS spectra of WSe_2_ before and after 4-NBD treatment. a,** W 4*f* core levels. **b,** Se 3*d* core levels. **c,** N 1*s* core level. The figures (a) and (b) are the unnormalized data from Figs. 4a and 4b in the main text. The spectra are shifted vertically for clarity.


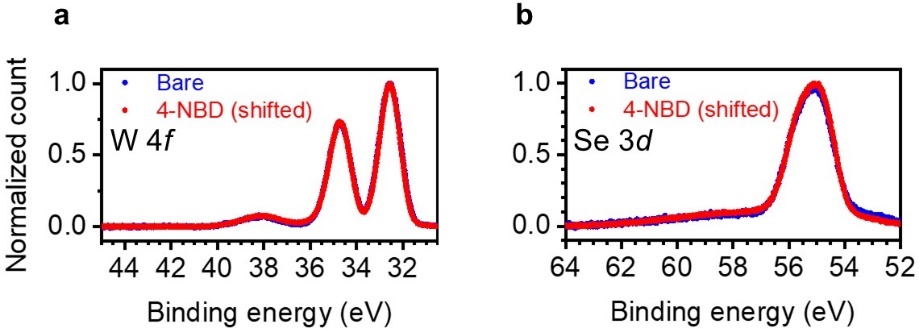


**Supplementary Fig. 15 | Superimposed W and Se core level XPS spectra before and after 4-NBD treatment.** The spectra are background-subtracted and normalized. The core level spectra after 4-NBD treatment are shifted to match the binding energy of the bare untreated sample. No noticeable changes are observed in the lineshapes of the W and Se core level features following the 4-NBD treatment.


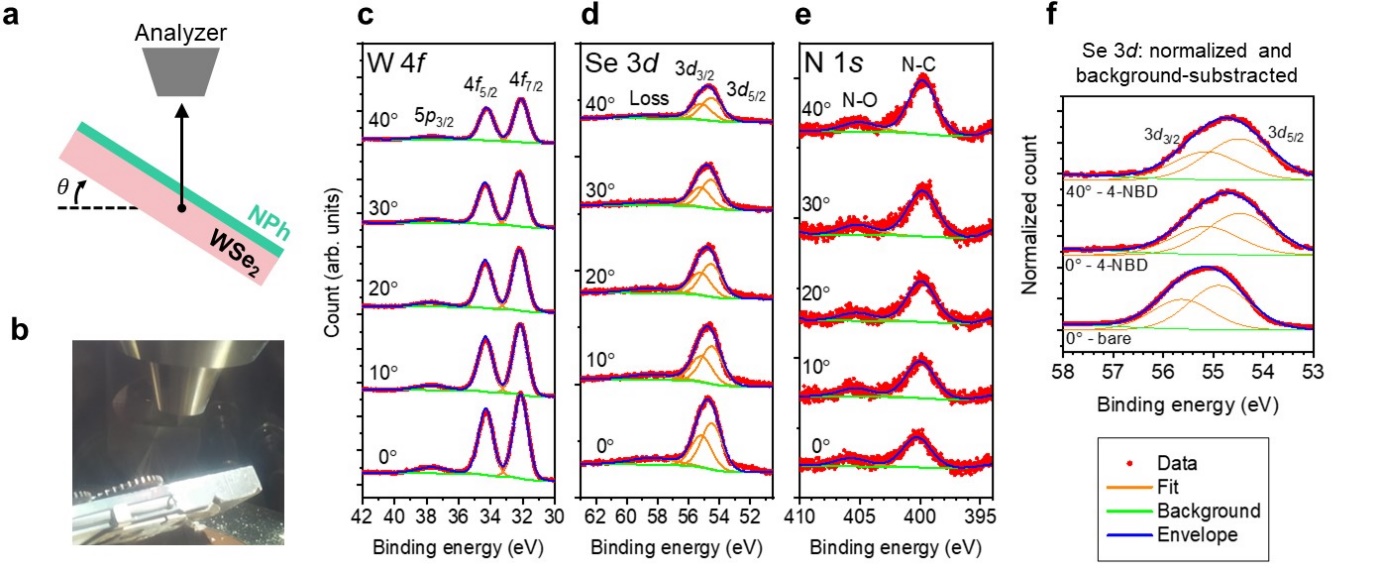


**Supplementary Fig. 16 | Angle-resolved XPS (ARXPS) measurements. a,** Schematic illustration of the ARXPS technique. The tilt angle *θ* is indicated. **b,** Photograph of the analysis chamber during the ARXPS measurement at *θ =* 40°. **c-e,** Spectra for **(c)** W 4*f*, **(d)** Se 3*d*, **(e)** N 1*s* core levels for tilt angles between 0° and 40°. **f,** Se 3*d* core level spectra before and after 4-NBD treatment at *θ =* 0° and 40° following background subtraction and count normalization. The spectra are shifted vertically for clarity.


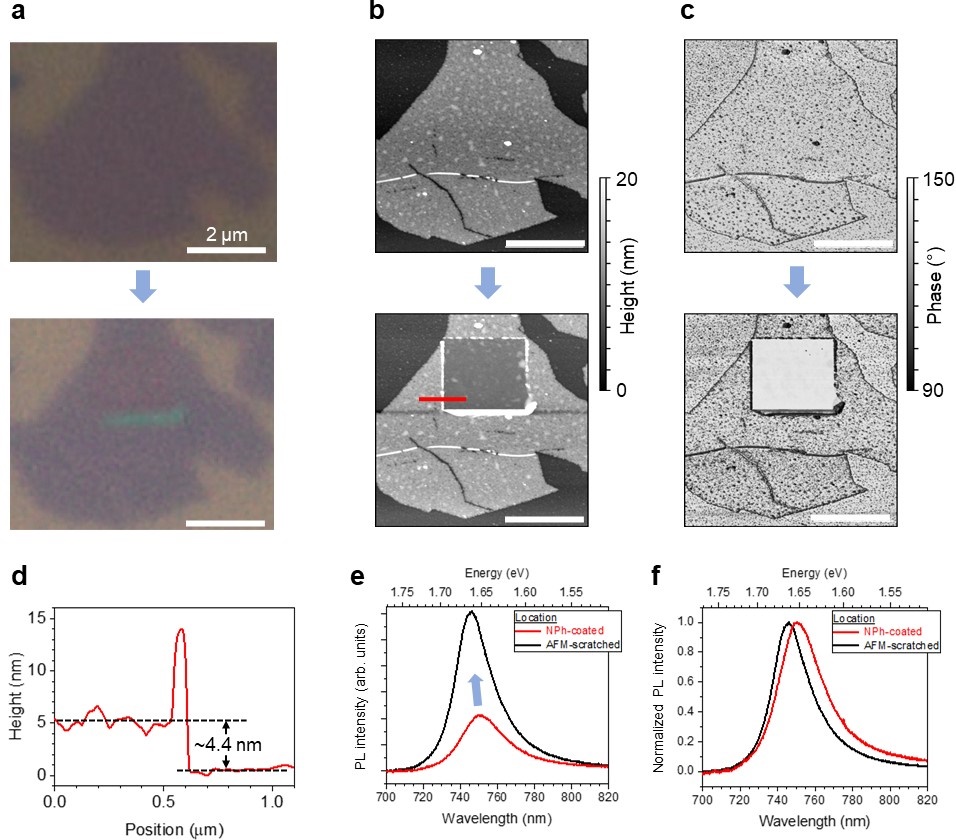


**Supplementary Fig. 17 | AFM scratching of a NPh film on 1L WSe­_2_ on a SiO_2_ substrate. a-c,** Comparison of 4-NBD-treated 1L WSe_2_ before scratching (upper row) and after AFM scratching (lower row). **a,** Bright-field optical micrograph. **b,** AFM height image. **c,** AFM phase image. The sample shows a 2×2 µm^2^ region where the NPh film is removed from the WSe_2_ surface following contact-mode AFM. All scale bars are 2 µm. **d,** Height profile measured along the red line in the AFM-scratched sample. The 4.4 nm thickness of the NPh film is determined from the step edge of the scratched area. **e,f,** PL spectra of the as-treated 1L WSe_2_ that remains coated with NPh film (red) and has been AFM-scratched to remove the NPh film (black). The spectra in (e) are collected from the PL map presented in Figs. 4d-f in the main text. After removal of the NPh film with AFM scratching, the PL intensity of 1L WSe_2_ increases significantly. Meanwhile, the spectra in (f) are normalized to emphasize the blueshift and sharpening of the spectrum following AFM scratching.


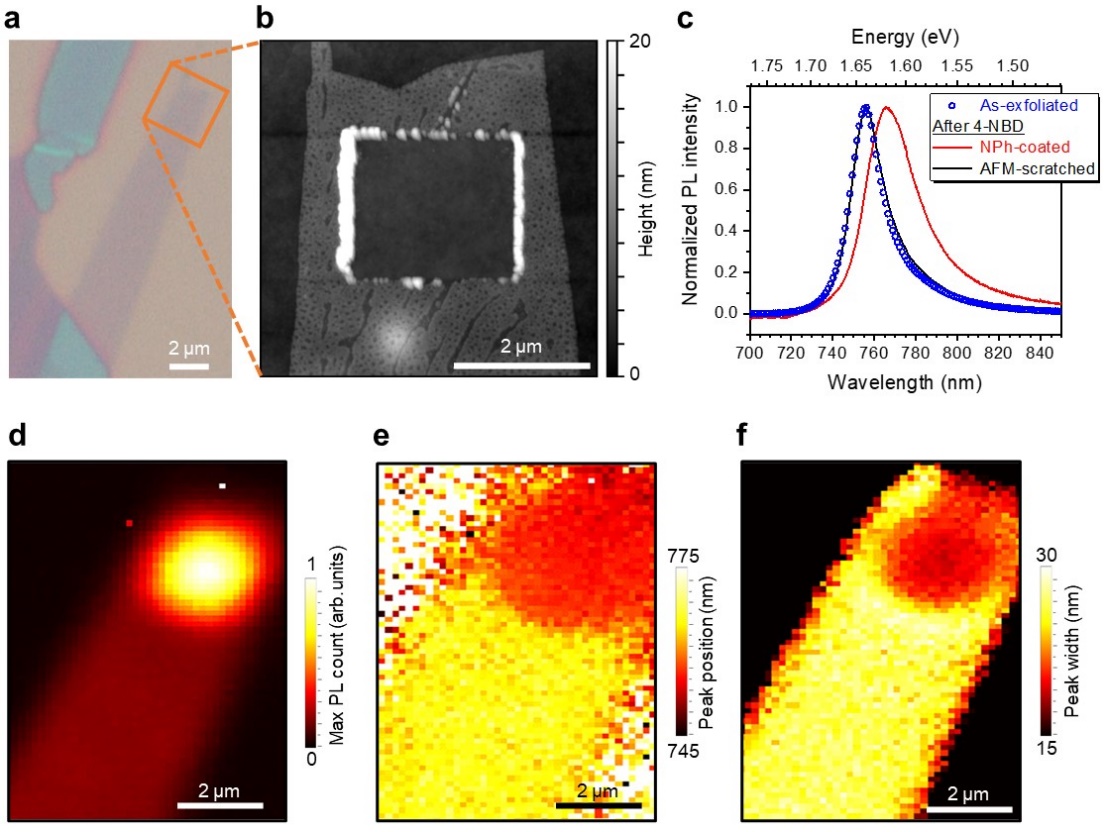


**Supplementary Fig. 18 | AFM scratching of NPh film on 1L WSe­_2_ on a PMMA-coated SiO_2_ substrate.** AFM scratching of NPh from WSe_2_ on a PMMA-coated SiO_2_ substrate can be performed at a low applied force setpoint. **a,** Optical micrograph. **b,** AFM height image of the sample following the scratching of the NPh film. **c,** Normalized PL spectra of the sample as-exfoliated on the substrate (blue circles), region with NPh coating after 4-NBD treatment (red line), and region with the NPh film removed following AFM scratching (black line). The lineshape of the spectrum after AFM scratching is nearly identical to that of the sample as-exfoliated before 4-NBD treatment. For clarity, the spectrum from the as-exfoliated sample (blue) is reduced by showing only 1 point for every 5 data points. **d-f,** PL mapping of the sample at ambient conditions showing **(d)** PL peak height, **(e)** peak position, and **(f)** peak width. The AFM scratched area exhibits increased PL intensity, PL blueshift, and reduced PL linewidth, thus reversing the effect of the 4-NBD treatment. Reversal of the quenching effect has also been reported in other noncovalently functionalized 2D materials after the removal of the molecular adlayer, such as in the dissolution of NiPc in a WSe_2_-NiPc heterostructure^10^.


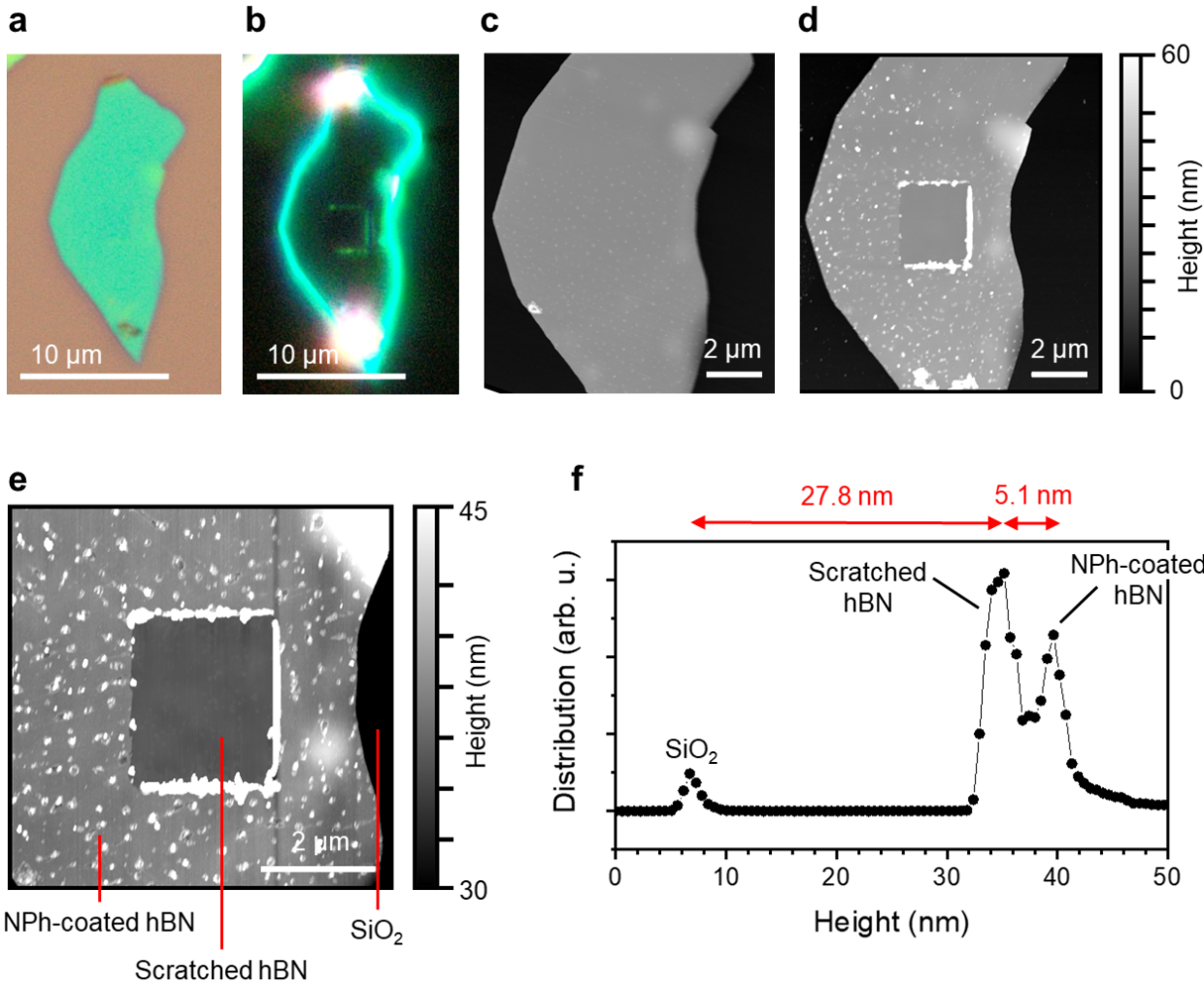


**Supplementary Fig. 19 | AFM scratching of a NPh film on hBN.** This experiment shows that the immersion of hBN into an aqueous solution of 4-NBD tetrafluoroborate can also result in NPh coating the surface of hBN. **a,b,** Optical micrographs of an hBN flake that has been treated with 4-NBD and AFM-scratched to remove the NPh film: **(a)** Bright-field and **(b)** dark-field images. **c,d,** AFM height images of the hBN flake **(c)** as-exfoliated and **(d)** after 4-NBD treatment and AFM-scratching. **e,** A smaller area AFM scan surrounding the AFM-scratched area. **f,** Height histogram of the pixels from the image in (e). The 3 peaks in the histogram are assigned to the SiO_2_ surface, AFM-scratched hBN, and NPh-coated hBN. The height differences indicate a thickness of 5.1 nm for the NPh film.


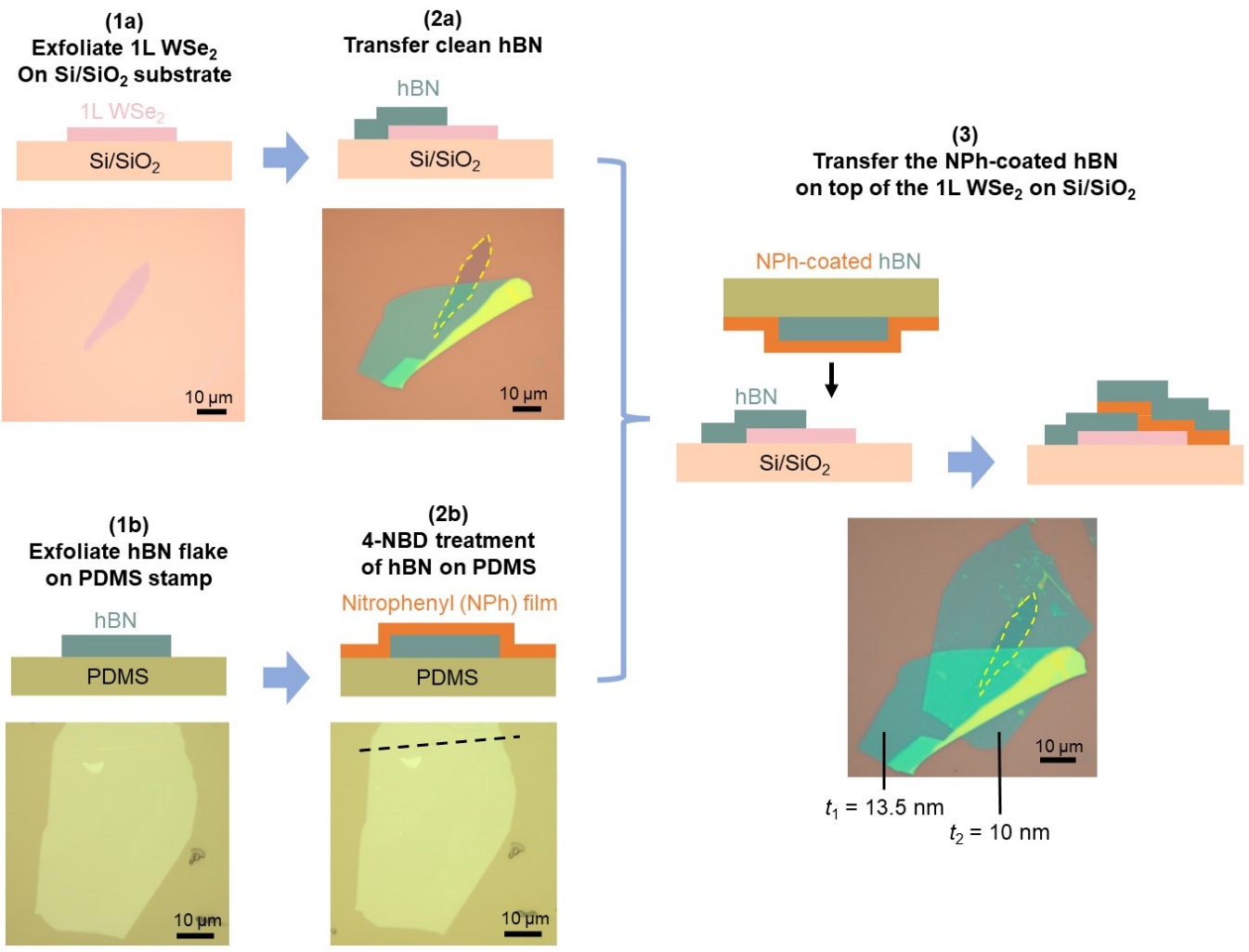


**Supplementary Fig. 20 | Procedure for hBN-assisted NPh film transfer.** The PL maps for this sample are presented in Figs. 4g-k in the main text. Optical micrographs of the sample at each step of the procedure are also presented. The thicknesses of the hBN flakes (*t*_1_: bottom hBN, *t*_2_: NPh-coated top hBN) as measured by AFM are also given. The hBN flake was torn along the black dashed line during the final transfer process.


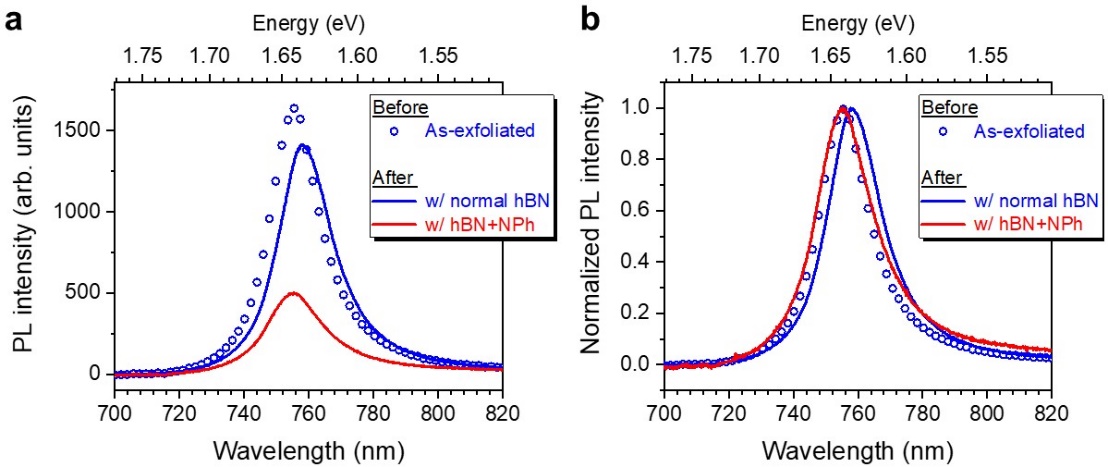


**Supplementary Fig. 21 | Photoluminescence of 1L WSe_2_ with hBN-assisted NPh film transfer.** The sample and PL map data are presented in Figs. 4g-k in the main text. **a,** PL spectra of the as-exfoliated sample (blue circles, Fig. 4j in the main text), sample region covered with normal hBN (blue line), and sample region contacted with transferred NPh (red line). **b,** The same data in (a) with normalized intensity. Unlike the NPh film coating from sample immersion in 4-NBD solution, the functionalization via hBN-assisted NPh film transfer does not produce a redshift or broadening of the PL spectrum.


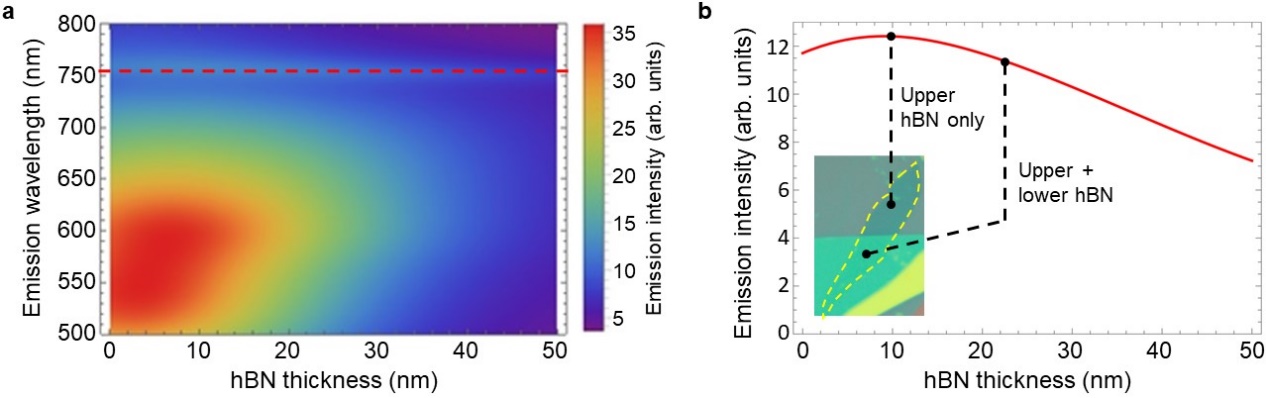


**Supplementary Fig. 22 | Simulation of light outcoupling from the multi-layered stack of WSe_2_ with hBN-assisted NPh transfer.** **A,** Light emission intensity from the sample as a function of emission wavelength and total hBN layer thickness (upper and lower hBN). **B,** A linecut of the colormap along the red dashed line in (a) that corresponds to the peak PL emission of WSe_2_ at 756 nm.

An exact model to determine the effect of total hBN thickness in the variation of interference and local field changes for sample in Fig. 4g-i is not possible because the complex refractive index of the NPh film is unknown. However, we can simplify the model by assuming that the effect of the nitrophenyl film on the overall reflectivity of multilayer film is negligible, which is reasonable given how the presence of the NPh film does not appear to change the color contrast of the WSe_2_ or hBN that it covers (e.g., Supplementary Figs 17a, 18a, 19a, and 20). This observation is likely due to the thinness (~4-5 nm) and relatively small refractive index of the film, which also indicates a small contribution of the optical phase shift through the NPh layer.

We then calculated the total intensity of light emission outcoupled from the sample following Ref. 11. Supplementary Fig. 22a shows the outcoupled light intensity as a function of the emission wavelength and the total thickness of the lower and upper hBN above the monolayer WSe_2_. Here, the excitation wavelength of 532 nm and bottom SiO_2_ thickness of 285 nm are used in the modelling. The refractive index of WSe_2_ is referred from Ref. 12 and is approximated by a series of Lorentz oscillators. The refractive index of hBN is obtained from Ref. 13.

Supplementary Fig. 22b shows a linecut of the colormap along the peak PL wavelength of WSe_2_ at ~756 nm. The calculated intensity curve is marked for values that correspond to the region of the WSe_2_ with direct contact to NPh film and that of the region with additional hBN capping. The calculation suggests that if there were no electronic coupling between NPh and WSe_2_, the PL of WSe_2_ with transferred NPh (upper half of the sample in Fig. 4i in the main text) would have appeared stronger than the PL from segment without direct NPh contact by a factor of ~1.1 due to the multilayer interference effect. Since this enhancement was not observed, our calculation confirms that the PL quenching effect from the transferred NPh film is compatible with the interpretation of a type-II heterojunction formation.


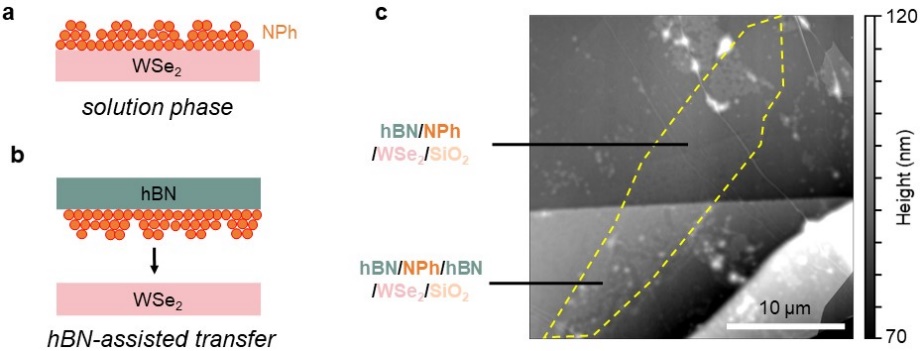


**Supplementary Fig. 23 | Explanation of weaker PL quenching effect in WSe_2_ with transferred hBN as compared to WSe_2_ with solution treatment of 4-NBD. a,** Illustration of WSe_2_ with NPh oligomer film from solution phase treatment of 4-NBD. Although the bottom NPh/WSe_2_ should be conformal, the top surface of NPh film typically appears as a rough film (e.g., AFM in Supplementary Figs. 17b, 18b, and 19e). **b,** Illustration of WSe_2_ with transferred NPh. **c,** AFM image of WSe_2_ with transferred NPh film (Fig. 4h in the main text). The topography of the monolayer WSe_2_ (marked with yellow dashed line) and the inhomogeneity of the transferred NPh film remains visible through the top hBN stack. The non-conformality of the WSe_2_/NPh interface in the transferred case leads to the weaker PL quenching effect in WSe_2_ with transferred hBN.

**
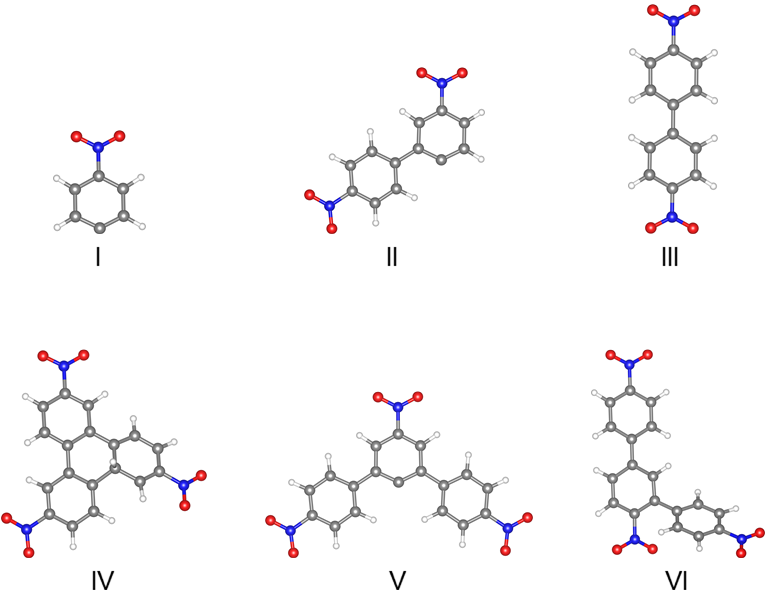
**

**Supplementary Fig. 24 | Structures of the NPh oligomers considered in the first-principles calculations.** (I) 1-ring NPh monomer, (II)-(III) 2-ring NPh oligomer, (IV)-(VI) 3-ring NPh oligomer. The Roman numerals I-VI will be used to refer to the corresponding structures in subsequent supplementary figures.


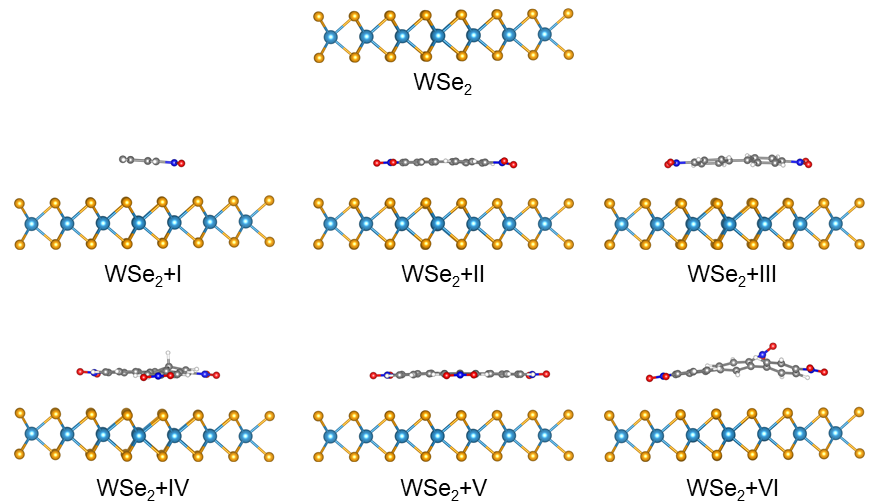


**Supplementary Fig. 25 |** **Side-view of the optimized structure of 1L WSe_2_ functionalized with NPh oligomers**. In the most stable configurations, which are represented here, the molecules are almost flat on the surface, and the smallest distance between the surface and molecule is ~3.5 Å. The Roman numerals refer to the structure of the NPh oligomers as depicted in Supplementary Fig. 24.


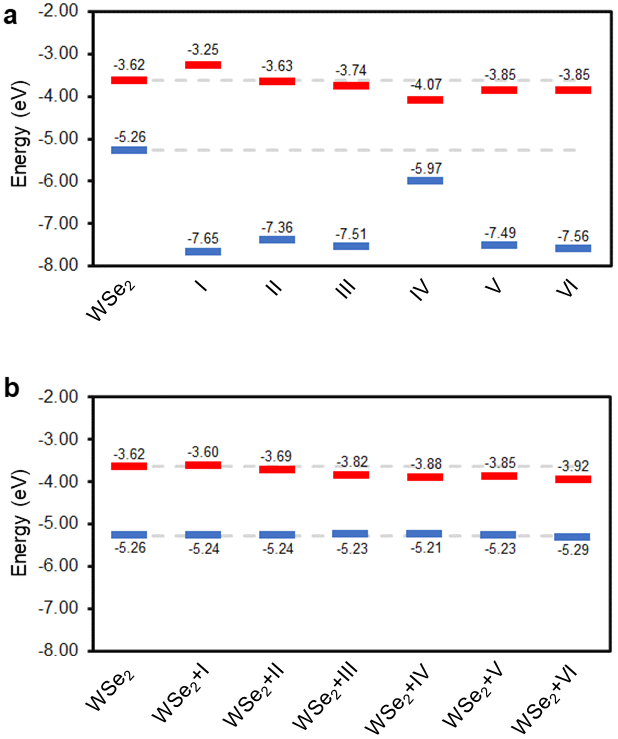


**Supplementary Fig. 26 | Energy-level alignment between 1L WSe_2_ and NPh.** **a,** The valence band maximum (VBM, blue bars) and conduction band minimum (CBM, red bars) of pristine 1L WSe_2_, and the HOMO/SOMO and LUMO of isolated NPh oligomers. **b,** Band edges of the hybrid WSe_2_-NPh structure after the contact of WSe_2_ and NPh. For pristine 1L WSe_2_ (left-most column) and WSe_2_+I configuration, the blue and red bars refer to the VBM and CBM of the 1L WSe_2_, respectively. For the rest of the configurations, the blue bars refer to the VBM of 1L WSe_2_, whereas the red bars refer to the LUMO levels of the oligomers after the WSe_2_-NPh contact. The vacuum level is set to 0 eV.

**
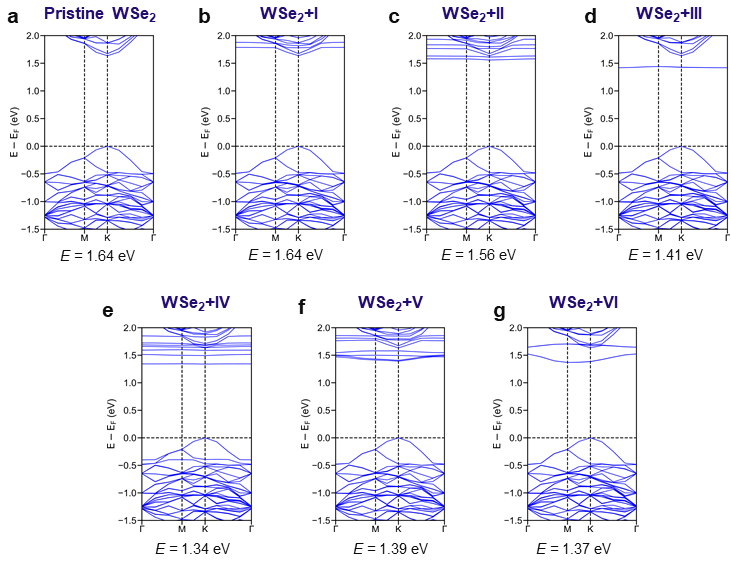
**

**Supplementary Fig. 27 | Electronic band structures of WSe_2_ and WSe_2_-NPh heterojunctions without strain. a,** Pristine 1L WSe_2_. **b,** WSe_2_+I (NPh monomer). **c,** WSe_2_+II. **d,** WSe_2_+III. **e,** WSe_2_+IV. **f,** WSe_2_+V. **g,** WSe_2_+VI. The calculation is based on the configurations shown in Supplementary Fig. 24. The Fermi energy is set to the valence band edge energy. The value of *E* under each band structure represents the energy of either the conduction band edge of 1L WSe_2_ or the LUMO level of the NPh oligomer (whichever is the lowest). The band structures reveal that the deposition of NPh oligomers on WSe_2_ leads to the appearance of semi-flat bands near the conduction band edge. The energy of these states depends on the number of rings in the oligomer. New mid-gap flat bands from the oligomer electronic orbitals are clearly present in (c) to (g), resulting in type-II mixed-dimensional heterojunctions between WSe_2_ and NPh. Meanwhile, we anticipate that the WSe_2_+I arrangement is not likely to dominate the functionalized film given the high reactivity of the open-shell structure and its tendency to oligomerize.

**Supplementary Table 2 | Binding energies of the oligomers with the WSe_2_ surface calculated with the PBE-D3BJ correction.** The binding energies reveal that among the 2-ring molecules (II and III), configuration II binds most strongly with the surface. These results indicate that configuration II is most stable in the WSe_2_-NPh heterojunction formation among the 2-ring molecules. Similarly, among the 3-ring molecules (IV, V and VI), V binds most strongly, indicating that configuration V is most stable in the WSe_2_-NPh heterojunction formation among the 3-ring molecules. Hence, in subsequent supplementary figures and the main text discussion, we focused primarily on cases II and V. Hereafter, structure II is denoted as *NPh (2-ring)*, and structure V is denoted as *NPh (3-ring)*.


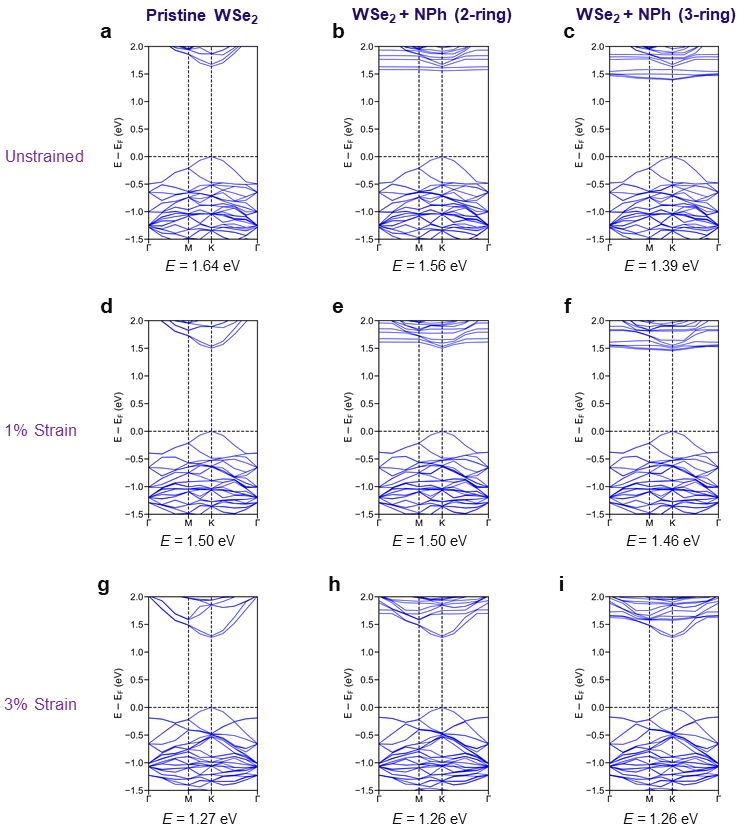


**Supplementary Fig. 28 | Electronic band structures of defect-free 1L WSe_2_ at different levels of biaxial tensile strain**. The band structures without functionalization, with NPh (2-ring), and with NPh (3-ring) are considered. **a-c,** Band structure without strain. **d-f,** Band structures with 1% biaxial tensile strain. **g-i,** Band structures with 3% biaxial tensile strain. The Fermi energy is set to the valence band edge. The value of *E* under each band structure represents the energy of either the conduction band edge of 1L WSe_2_ or the LUMO level of the NPh oligomer (whichever is the lowest). The band structures reveal that the energies of the semi-flat mid-gap bands from the NPh oligomer electronic states near the WSe_2_ conduction band edge remain almost unchanged regardless of the strain applied to WSe_2_. Meanwhile, the dispersive conduction bands from 1L WSe_2_ are lower in energy when the strain is applied. Additionally, more overlap between the oligomer orbitals and the WSe_2_ bands occurs at 3% strain.


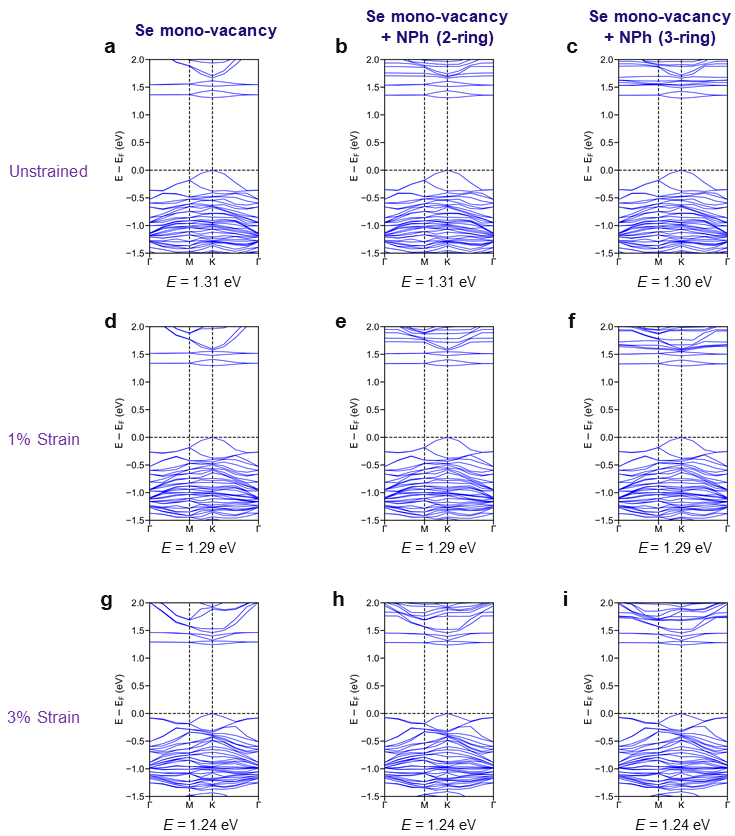


**Supplementary Fig. 29 | Electronic band structures of 1L WSe_2_ with a Se mono-vacancy at different levels of biaxial tensile strain**. The band structures for sample configurations without functionalization, with NPh (2-ring), and with NPh (3-ring) are considered. **a-c,** Band structures without strain. **d-f,** Band structures with 1% biaxial tensile strain. **g-i,** Band structures with 3% biaxial tensile strain. The Fermi energy is set to the valence band edge. The value of *E* under each band structure represents the energy minimum of the Se mono-vacancy defect states relative to the Fermi energy. These lowest mid-gap defect bands are predominantly localized on the three W atoms surrounding the Se vacancy.

**
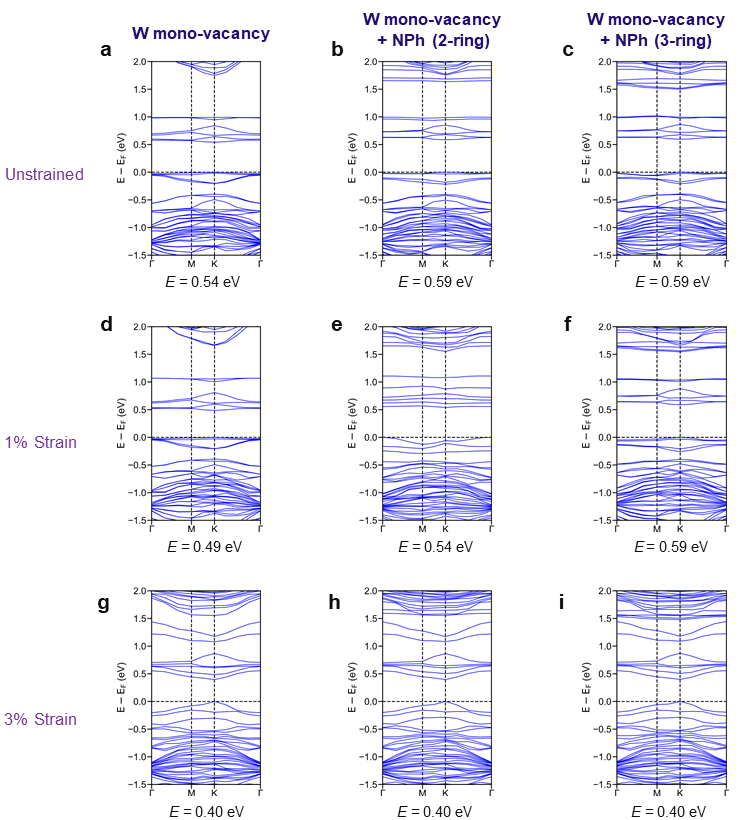
**

**Supplementary Fig. 30 | Electronic band structures of WSe_2_ with a W mono-vacancy at different levels of biaxial tensile strain.** The band structures for configurations without functionalization, with NPh (2-ring), and with NPh (3-ring) are considered. **a-c,** Band structures without strain. **d-f,** Band structures with 1% biaxial tensile strain. **g-i,** Band structures with 3% biaxial tensile strain. The Fermi energy is set to the valence band edge. The value of *E* under each band structure represents the energy minimum of the W mono-vacancy defect states relative to the Fermi energy. With the W mono-vacancy, there is the appearance of additional low-lying mid-gap states around 0.4 eV to 1.0 eV in addition to changes in the valence bands of WSe_2_. In real space, the new mid-gap states are localized either on the W atomic plane near the defect site or coupled between the Se and W near the vacancy.


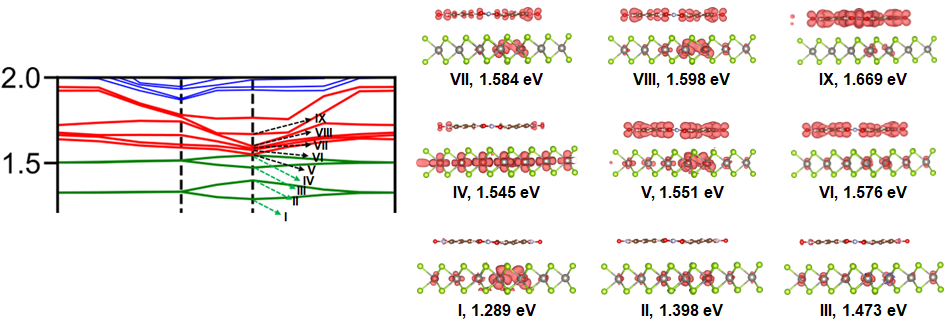


**Supplementary Fig. 31 | Real-space distribution of the density of states at the K point for Se vacancies, NPh orbitals, and conduction band edges.** The calculated band structure shown in Fig. 5d of the main text shows electronic coupling between the NPh orbitals and the uppermost vacancy state that facilitates quenching of the defect emission. The results in this figure are based on the band structure calculation shown in Fig. 5d. The band labelled as “V” shows significant density of states distribution in real-space at both the NPh oligomer and the Se vacancy.


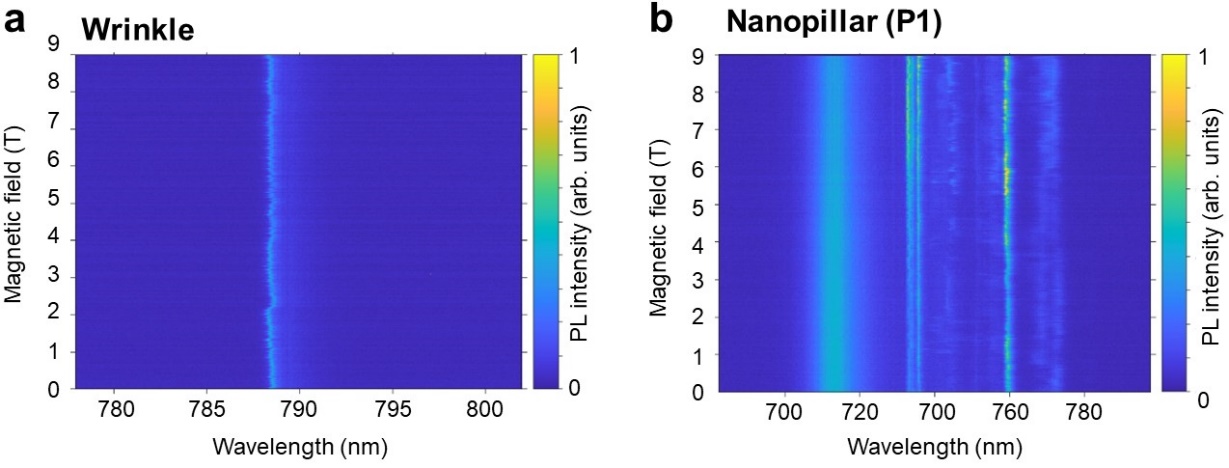


**Supplementary Fig. 32 | Magnetic field dependence of the emission from the chemomechanically-modified emitters.** The magnetic field is applied in the out-of-plane direction from the substrate. **a,** SPE in wrinkled 1L WSe_2_ from Fig. 2 in the main text. **b,** SPE from nanopillar P1 from Fig. 3 in the main text. In both cases, the SPE peaks do not show measurable shifting, splitting, or broadening. Following Refs. ^6,14^, SPEs that do not show a Zeeman shift or splitting with magnetic field may be attributed to transitions between states with the same *g*-factors.

**Supplementary References**

1. Xu H., Wang Q. Cucurbit[7]uril/CuCl promoting decomposition of 4-nitrobenzenediazonium in aqueous solution. *Chin. Chem. Lett.* **30**, 337-339 (2019).

2. Palacios-Berraquero C., et al. Large-scale quantum-emitter arrays in atomically thin semiconductors. *Nat. Commun.* **8**, 15093 (2017).

3. Tonndorf P., et al. Single-photon emission from localized excitons in an atomically thin semiconductor. *Optica* **2**, 347-352 (2015).

4. Chakraborty C., Kinnischtzke L., Goodfellow K. M., Beams R., Vamivakas A. N. Voltage-controlled quantum light from an atomically thin semiconductor. *Nat. Nanotechnol.* **10**, 507-511 (2015).

5. Srivastava A., Sidler M., Allain A. V., Lembke D. S., Kis A., Imamoğlu A. Optically active quantum dots in monolayer WSe_2_. *Nat. Nanotechnol.* **10**, 491-496 (2015).

6. He Y.-M., et al. Single quantum emitters in monolayer semiconductors. *Nat. Nanotechnol.* **10**, 497-502 (2015).

7. Rosenberger M. R., et al. Quantum calligraphy: writing single-photon emitters in a two-dimensional materials platform. *ACS Nano* **13**, 904-912 (2019).

8. Montblanch A. R. P., et al. Confinement of long-lived interlayer excitons in WS_2_/WSe_2_ heterostructures. *Commun. Phys.* **4**, 119 (2021).

9. Yong C.-K., et al. Valley-dependent exciton fine structure and Autler–Townes doublets from Berry phases in monolayer MoSe_2_. *Nat. Mater.* **18**, 1065-1070 (2019).

10. Choi J., Zhang H., Choi J. H. Modulating optoelectronic properties of two-dimensional transition metal dichalcogenide semiconductors by photoinduced charge transfer. *ACS Nano* **10**, 1671-1680 (2016).

11. Lien D.-H., et al. Engineering light outcoupling in 2D materials. *Nano Lett.* **15**, 1356-1361 (2015).

12. Jung G.-H., Yoo S., Park Q.-H. Measuring the optical permittivity of two-dimensional materials without a priori knowledge of electronic transitions. *Nanophotonics* **8**, 263-270 (2019).

13. Lee S.-Y., Jeong T.-Y., Jung S., Yee K.-J. Refractive index dispersion of hexagonal boron nitride in the visible and near-infrared. *Phys. Stat. Sol. b* **256**, 1800417 (2019).

14. Müller T., et al. Wide-range electrical tunability of single-photon emission from chromium-based colour centres in diamond. *New J. Phys.* **13**, 075001 (2011).
